# DEVELOPMENT OF AN AI-POWERED EMOTIONALLY INTELLIGENT, INTERACTIVE AGENT PROTOTYPE TO SUPPORT INFORMAL CAREGIVERS

**DOI:** 10.1093/geroni/igad104.3274

**Published:** 2023-12-21

**Authors:** Yu-Ping Chang, Aditya Kumar Dwibedi, Suryakanth Joshi, Sreyasee Das Bhattacharjee

**Affiliations:** University at Buffalo, The State University of New York, Buffalo, New York, United States; University at Buffalo, The State University of New York, Buffalo, New York, United States; University at Buffalo, The State University of New York, Buffalo, New York, United States; University at Buffalo, The State University of New York, Buffalo, New York, United States

## Abstract

Long-term informal caregiving for older adults with dementia is exhausting and poses risks to caregivers’ health outcomes. Most informal caregivers are not well prepared for their new role, facing various challenges and receiving limited support. With the advent of artificial intelligence (AI) and information and communication technologies (ICTs), assistive technology is increasingly useful for supporting caregivers. However, limited research assesses the potential for AI to facilitate a privacy-preserving computation environment to provide proactive assistance to caregivers. This project aims to fill this gap by leveraging the tremendous potential of Multimodal Machine Learning and Large Language Models (LLMs) to develop a prototype of an AI-powered emotionally intelligent, interactive agent for informal caregivers. We introduce an effective context-driven instruction tuning technique that enables LLMs to finetune various caregiving-related tasks (tips generation, answering questions, elaborating a specific training module) with instructions. The project will also deliver a first-of-its-kind custom dataset augmented with manualized instructions for relevant caregiving tasks, which is used for finetuning several state-of-the-art LLM models. Our intelligent interactive agent prototype currently has the ability to evaluate caregivers’ unique affective states at query times to provide personalized assistance, addressing various types of caregivers’ situation-specific queries (e.g., managing agitation, wandering, or medications). The prototype is capable of understanding and responding to the user’s natural language queries and designing short, machine-driven persuasive conversations on a pre-defined set of topics. Our prototype has the potential to provide individualized and contextualized support for caregiving tasks which in turn may greatly reduce caregiver burden.

